# Clinical evaluations of diaphysis malignant tumors of femur and tibia treated with microwave ablation in situ

**DOI:** 10.1186/s13018-020-01662-1

**Published:** 2020-04-09

**Authors:** Zhe Yu, Chuan Dong, Minghua Zhang, Tongshuan Gao, Rui Ding, Yindi Yang, Qingyu Fan

**Affiliations:** Department of Orthopedic Surgery, Orthopedics Oncology Institute of Chinese PLA, Tangdu Hospital, Air Force Military Medical University, 569 Xinsi Road of Baqiao District, Xi’an, 710038 Shaanxi People’s Republic of China

**Keywords:** Limb salvage surgery, Diaphysis, Bone tumor, Microwave ablation, Functional evaluation

## Abstract

**Background:**

This study was designed to evaluate the clinical outcomes of patients with diaphysis malignant tumors of femur and tibia treated with microwave ablation (MWA) in situ.

**Methods:**

Retrospective study of 32 patients with diaphysis malignant bone tumors of femur or tibia have been treated by microwave ablation. Instead of en bloc resection, hyperthermia ablation in situ was carried out followed by strengthen procedure. The patients were followed up for a period ranging from 36 to 180 months.

**Results:**

Twenty-five patients survived over 3 years and all of the patients alive have a satisfactory functional and cosmetic limb. The postoperative survival rate of MWA group was significantly higher than the amputation group in consecutive inclusions.

**Conclusions:**

MWA is a feasible and effective surgical method for limb salvage operation and it might offer an innovative and distinctive therapeutic alternative for diaphysis malignant bone tumors, which avoiding osteotomy or prosthesis replacement.

**Level of evidence:**

Level IV, clinical cohort study

## Introduction

Primary malignant tumors arising from the diaphysis of the weight-bearing long tubular bone, such as femur or tibia, are relatively uncommon compared to those occur inmetaphyseal region of long bones. Despite its lower incidence (less than 10% in all bone malignant tumor cases), the therapeutical strategy for the diaphysis malignant bone tumor remains challenging among surgeons of bone oncology [1]. Wide resection and limb salvage surgery were considered as the standard surgical procedure for malignant musculoskeletal tumors [2, 3]. Compared with simple amputation, limb salvage technique did not compromise survival rate. In contrast, it achieved a lower rate of local recurrence and partially retained the physical function of affected limbs [4]. Most patients with primary bone tumor are children and young adults; thus, the aim of treatment is supposed to not only excise the tumor site, prolong the patients’ life span, but also preserve the affected limbs, maintain the physical function without major complications or recurrences over long time.

Several materials could be applied to reconstruct the defect left after intercalary resection, such as intercalary prostheses [2] and bone allografts [5]. Autologous bone allograft includes vascularized autologous fibula grafts [6], extracorporeal irradiated and re-implanted cemented autologous bone graft. Unfortunately, there is still no consensus among orthopedic surgeons to reconstructed defects after wide resection, because each aforementioned procedure has its benefits and weaknesses; patients after prosthesis replacement could promptly restored to a nearly normal daily life but long-term loosening, wear, and breakage could also be seen for it; the considerable durability is the main advantage of biological allograft-based reconstruction, but the triad complication “infection, fracture, and nonunion,” which could occur after allograft procedures were frequently reported by other researchers [2, 7].

Microwave ablation is a kind of promising technique for medical use. When target tissues were radiated with electromagnetic wave, water and other polar molecules oscillate and these molecules can generate heat by frictional interaction between each other in all biological organisms. The intensive heating (with the temperature of 80–100 °C for 20 min) can cause all the living tissues to be irreversible devitalized with death of cells and disruption of the extracellular matrix. So far, the MWA procedure has been used as a classical hyperthermia ablation method among various cancer treatments with the intention to induce direct killing of tumor cells or modulation of tumor architecture [8]. According to our experiences, the MWA procedure was widely implemented as a preferred routine surgical procedure for extremity and pelvis malignant musculoskeletal tumors for decades [9].

## Methods

This was a single-centered and retrospective study approved by the Institutional Review Board of the Air Force Military Medical University Second Affiliated Hospital (Tangdu Hospital). We performed a retrospective review of all patients who diagnosed as diaphysis malignant bone tumors of femur or tibia of from May 2004 to October 2013. The inclusive criteria of this study were as follows: (1) patients who were diagnosed as a primary malignant bone tumor occurring diaphysis of femur and tibia confirmed by experienced pathologists, (2) patients who do not received any surgical procedures before coming to our institute, and (3) patients who signed the informed consent that all clinical data could be obtained for study using. If the patients were too young (< 8 years) or too old (> 70 years), they were not allowed to participate in this clinical research. In addition, the patients with preoperative multiple organ metastases (≥ 2 locations) were also excluded from this study.

### Patients

Among the inclusion, 42 patients were performed with microwave ablation, as a definite measure of limb-salvage procedure. Despite 10 patients who had been lost of follow-up, 32 patients were followed up for a period ranging from 36 to 180 months (averaging 56.69 ± 38.71 months). Another 32 patients who accepted amputation treatment were selected as contrastive cases. All patients were evaluated for the clinical and functional outcomes by musculoskeletal tumor society (MSTS) rating scales. The demographic data of 32 patients treated with MVA and 32 patients treated with amputation were presented as Table 1 and Table 2. The mean age of the patients at the time of surgery was 30.94 ± 18.46 years (ranging from 11 to 66). All cases had a histological diagnosis based on incisional biopsy (Table 3). Of the 64 cases (30 men and 34 women), the most common tumor was osteosarcoma (24 cases) followed by metastatic neoplasm (16cases); the femur was the most frequently involved skeletal site (26 cases) followed by the tibia (6 cases).

**Table 1:** Details of patients who suffered from diaphysis malignant tumors of femur/tibia and underwent microwave ablation

Case	Gender/age (year)	Location	Stage^a^	Histology	Pathological fracture	Length (cm)	Ablation time (min)	MSTS score, (%)	Outcome	Follow-up (months)
1	M/15	DF (L)	IIB	OSA	N	10	30	97	A	180
2	M/17	MF (R)	IIB	OSA	N	12	30	100	A	36
3	F/14	MF (L)	IIB	OSA	N	8	30	97	A	120
4	F/54	DT (L)	IIIB	MN (thyroid)	N	5	30	97	A	48
5	M/17	DF (L)	IIB	OSA	N	12	30	93	A	132
6	F/58	DF (R)	IIIB	MN (renal)	Y	8	30	97	A	36
7	M/50	PF (L)	IIIB	MN (renal)	N	6	30	93	A	60
8	M/15	DF (R)	IIB	OSA	N	15	30	97	A	132
9	M/61	DF (R)	IIIB	MN (prostate)	N	5	30	87	A	84
10	M/18	MF (L)	IIB	OSA	N	15	30	90	A	60
11	F/16	MF (R)	IIB	EWS	N	9	30	90	A	84
12	F/11	MF (L)	IIB	EWS	N	7	30	87	A	96
13	F/23	PF (R)	IIB	CHOS	N	15	30	93	A	72
14	F/45	MF (R)	IIIB	MN (breast)	N	6	30	93	A	48
15	F/66	PF (L)	IIIB	MN (thyroid)	N	5	30	83	D	28
16	M/43	PF (L)	IIB	CHOS	N	4	30	90	A	48
17	M/64	MF (L)	IIB	IGTB	Y	15	30	100	A	36
18	M/43	PF (L)	IIB	CHOS	N	20	30	93	A	60
19	M/25	PF (L)	IIB	CHOS	N	10	30	97	A	48
20	F/61	PF (R)	IIIB	MN (renal)	Y	8	30	87	D	21
21	F/42	PF (R)	IIB	SSA	N	8	30	93	A	36
22	M/17	DF (R)	IIB	OSA	N	7	30	73	D	20
23	M/17	PT (R)	IIB	OSA	N	9	30	97	A	36
24	F/16	MT (R)	IIB	ABA	N	12	20	97	A	36
25	F/16	PT (R)	IIB	EWS	N	8	30	90	A	36
26	M/48	PT (R)	IIIB	MN (liver)	N	6	30	87	D	16
27	F/18	DF (R)	IIB	OSA	N	8	30	83	D	34
28	M/12	MF (L)	IIB	OSA	N	8	30	100	A	60
29	F/32	MF (L)	IIB	CHOS	N	15	30	93	A	36
30	M/18	DF (R)	IIB	OSA	Y	10	30	97	A	36
31	F/16	PT (L)	IIB	OSA	N	12	30	87	D	24
32	F/22	MF (R)	IIB	OSA	N	10	30	90	D	15

*PF* proximal femur, *MF* middle femur, *DF* distal femur, *PT* proximal tibia, *MT* middle tibia, *DT* distal tibia, *L* left, *R* right, *OSA* osteosarcoma, *CHOS* chondrosarcoma, *EWS* Ewing’s sarcoma, *IGTB* invasive giant cell tumor of bone, *SSA* synovial sarcoma, *ABA* ameloblastoma, *MN* metastatic neoplasm, *A* alive, *D* death, *Y* yes, *N* no, *MSTS* musculoskeletal tumor society, ^a^Enneking surgical stage

**Table 2:** Details of patients who suffered from diaphysis malignant tumors of femur/tibia and underwent amputation

Case	Gender/age (year)	Location	Stage	Histology	Pathological fracture	Length (cm)	MSTS score, (%)	Outcome	Follow-up (months)
1	M/14	DF (R)	IIIB	OSA	Y	12	60	D	11
2	F/9	MT (R)	IIB	OSA	N	10	80	D	18
3	M/21	MF (L)	IIB	EWS	N	14	77	A	48
4	F/76	DT (R)	IIIB	MN (lung)	N	6	83	D	23
5	F/58	DT (L)	IIIB	MN (breast)	N	8	77	D	32
6	M/28	PF (R)	IIB	MFCT	N	12	50	D	26
7	M/8	PT (L)	IIB	EWS	N	6	73	D	19
8	F/15	DF (L)	IIB	OSA	Y	12	63	D	36
9	M/16	PT (R)	IIIB	OSA	N	12	80	D	12
10	M/38	MF (R)	IIB	CHOS	N	18	80	A	48
11	F/14	PF (L)	IIB	CHOS	N	20	83	A	96
12	F/53	DF (L)	IIIB	MN (renal)	Y	8	87	A	60
13	F/43	MF (R)	IIIB	MM	Y	7	80	A	48
14	M/42	DF (R)	IIB	OSA	N	10	87	A	36
15	F/46	DF (L)	IIB	MFCT	N	8	63	D	17
16	M/53	DF (R)	IIB	IGTB	Y	10	83	A	60
17	M/44	PF (L)	IIB	CHOS	N	15	70	A	36
18	F/13	PT (L)	IIB	OSA	N	10	77	A	60
19	F/65	MF (R)	IIIB	MN (lung)	Y	10	57	D	9
20	M/8	DF (R)	IIIB	OSA	N	6	80	D	10
21	F/41	MT (L)	IIB	ABA	Y	10	93	A	36
22	M/57	MF (R)	IIIB	MN (liver)	N	5	70	D	21
23	F/16	PT (L)	IIB	EWS	N	8	80	D	16
24	M/12	DF (R)	IIB	OSA	N	12	73	D	12
25	M/38	PF (R)	IIB	CHOS	N	13	60	A	60
26	F/58	MT (R)	IIIB	MN (breast)	N	7	77	D	18
27	F/61	DF (L)	IIIB	MN (renal)	Y	6	83	A	36
28	F/14	PT (L)	IIB	OSA	N	10	90	A	72
29	F/42	MT(R)	IIIB	MN (breast)	Y	8	93	A	36
30	M/15	DF (L)	IIB	OSA	N	12	77	A	48
31	F/17	DF (R)	IIB	OSA	Y	10	67	D	14
32	F/46	MF (R)	IIIB	MFCT	N	12	73	D	10

*PF* proximal femur, *MF* middle femur, *DF* distal femur, *PT* proximal tibia, *MT* middle tibia, *DT* distal tibia, *L* left, *R* right, *OSA* osteosarcoma, *CHOS* chondrosarcoma, *EWS* Ewing’s sarcoma, *IGTB* invasive giant cell tumor of bone, *ABA* ameloblastoma, *MN* metastatic neoplasm, *MFCT* malignant fibrous cell tumors, *MM* multiple myeloma, *A* alive, *D* death, *Y* yes, *N* no, *MSTS* musculoskeletal tumor society, ^a^Enneking surgical stage

**Table 3:** Histological diagnosis summary of diaphysis malignant tumors of femur/tibia after microwave ablation

Histological diagnosis	Location (pathological fracture)		No. of patients (*n* = 32)
	Femur	Tibia	
OSA osteosarcoma	11 (1)	2 (0)	13
CHOS chondrosarcoma	5 (0)	0 (0)	5
EWS Ewing’s sarcoma	2 (0)	1 (0)	3
IGTB invasive giant cell tumor of bone	1 (1)	0 (0)	1
SSA synovial sarcoma	1 (0)	0 (0)	1
ABA ameloblastoma	0 (0)	1 (0)	1
MN metastatic neoplasm	6 (2)	2 (0)	8

The primary tumor was evaluated on plain radiographs, computed tomography (CT) scans, and magnetic resonance imaging scans. The CT scanning of the chest and ultrasonic examination of abdominal organs were also performed to confirm that there were no newly occurring metastases.

For patients with osteosarcoma, Ewing’s sarcoma or other primary/metastasis highly malignant bone tumor, neoadjuvant chemotherapy before operation was given according to the clinical therapeutic guideline or authoritative literature documents. In detail, all the selected patients received the 2–3 circles of standard three-course neoadjuvant chemotherapy with a 4-week interval between cycles. After receiving the full course of neoadjuvant chemotherapy, all the patients were restaged using MRI and received surgery 3 weeks after the last course. Postoperative chemotherapy was performed every 3 months and lasted for 12 to 24 months.

### Surgical procedure

The main concept here is to dissect the tumor-bearing bone from surrounding normal tissues with a safe margin and subsequently perform an en bloc ablation using antenna-guided hyperthermia therapy. Currently, co-axial microwave antennas (require circulating water to cool the tip of the antenna to avoid metal melting damage due to high temperature) were inserted into the carefully dissected and isolated tumor tissue block. Three to 8 antennae were used variably depended on the tumor size and extension. The distance between two antennas was about 3 cm. The goal of thermal ablations was to create an ablation zone that extends at least 1 cm beyond the tumor boundary at all points after being heated to 70–100 °C for 20–30 min. During surgery, multiple thermocouples were placed in various critical locations to monitor the temperature within and around the bulk. The soft dead tissues were removed and/or curetted leaving behind the defective bone (bone scaffold with adequate strength) for reconstruction using any of the currently accepted methods, such as fibular autograft, allograft, or bone cement.

### Follow-up

Patients in both MVA and amputation groups were followed up for a period ranging from 36 to 180 months (mean, 57.1 months). The length of surgery and the duration of hospital stay were recorded for each case. Postoperative plaster immobilization was applied for 6 months and then removed. Patients were encouraged to do functional training with initial protection of the brace. At the 6-month postoperative follow-up and the subsequent follow-up visits, patients were asked to attend an outpatient clinic where a survey of the MSTS was registered to assess patients’ function and satisfaction. Postoperative A-P and lateral X-rays of the diseased region and the adjacent joints were taken at month 3, month 6, month 12, and every 6 months subsequently until bony union occurred, and then every 6 months until 5 years after the operation. The types of ablation time were noted in each case. Complications requiring further surgery were also recorded.

### Statistical analysis

Limb function was evaluated with the MSTS rating scales, which comprise six items, namely, pain, function, emotional acceptance, support, walking, and gait. Five points are allocated to each item and the highest score is 30 (100 %). Survival percentage of patients was recorded and analyzed using the log-rank (Mantel-Cox) test with 95% confidence interval. SPSS17.0 was used for data variation analysis. The mean and the standard deviation for baseline descriptive characteristics were calculated.

## Results

### Typical procedure and approaches based on our experiences

**Fig. 1:**
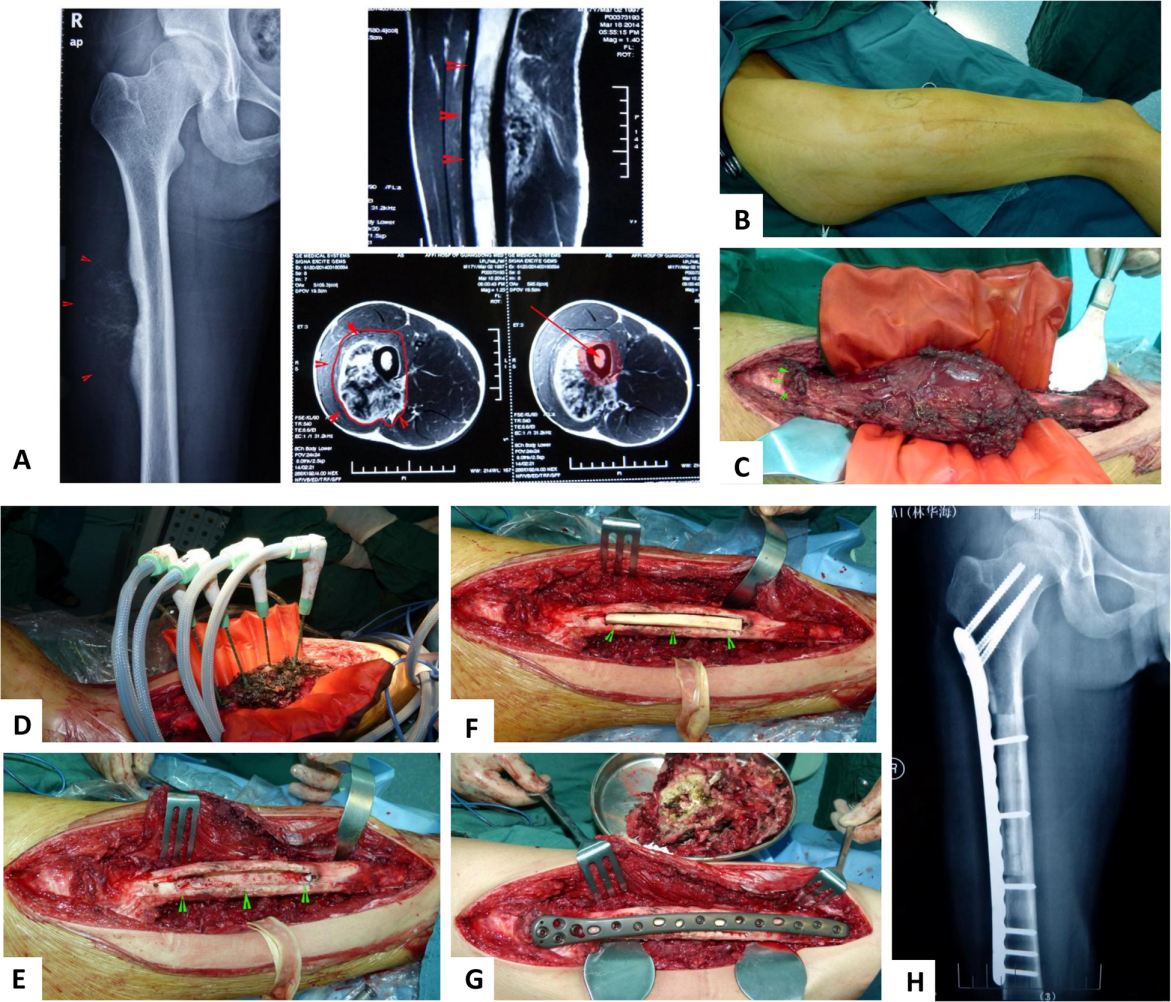
Typical procedure for malignant bone tumors at femoral diaphysis. **a** Image data of A-P X ray and MRI films demonstrated an osteosarcoma at femoral diaphysis before surgery. There were distinct signs of cortical bone destruction in the middle part of the right femoral shaft and visible soft tissue mass shadow in the anterolateral range of quadriceps femoris. **b** The incision demonstration from lateral thigh after skin degerming. **c** Dissect the tumor-bearing bone from surrounding normal tissues with safe margin and a heat-isolation pad was put between the tumor bone and surrounding normal tissues. **d** The microwave generator and antenna were inserted into the tumor bulk and began to deliver electromagnetic energy into the tumor bone. **e** Soft tumor mass and tumor bone inside the femoral diaphysis were removed and bone graft cavity was prepared. **f** The mixture materials of autologous fibular graft with allograft bone chips or bone cement were used for filling the cavity. **g** Restore the normal shape of the femoral diaphysis and give a prophylactic fixation. **h** Postoperative A-P X ray film showed the complete tumor removal and perfect bone transplantation

**Fig. 2:**
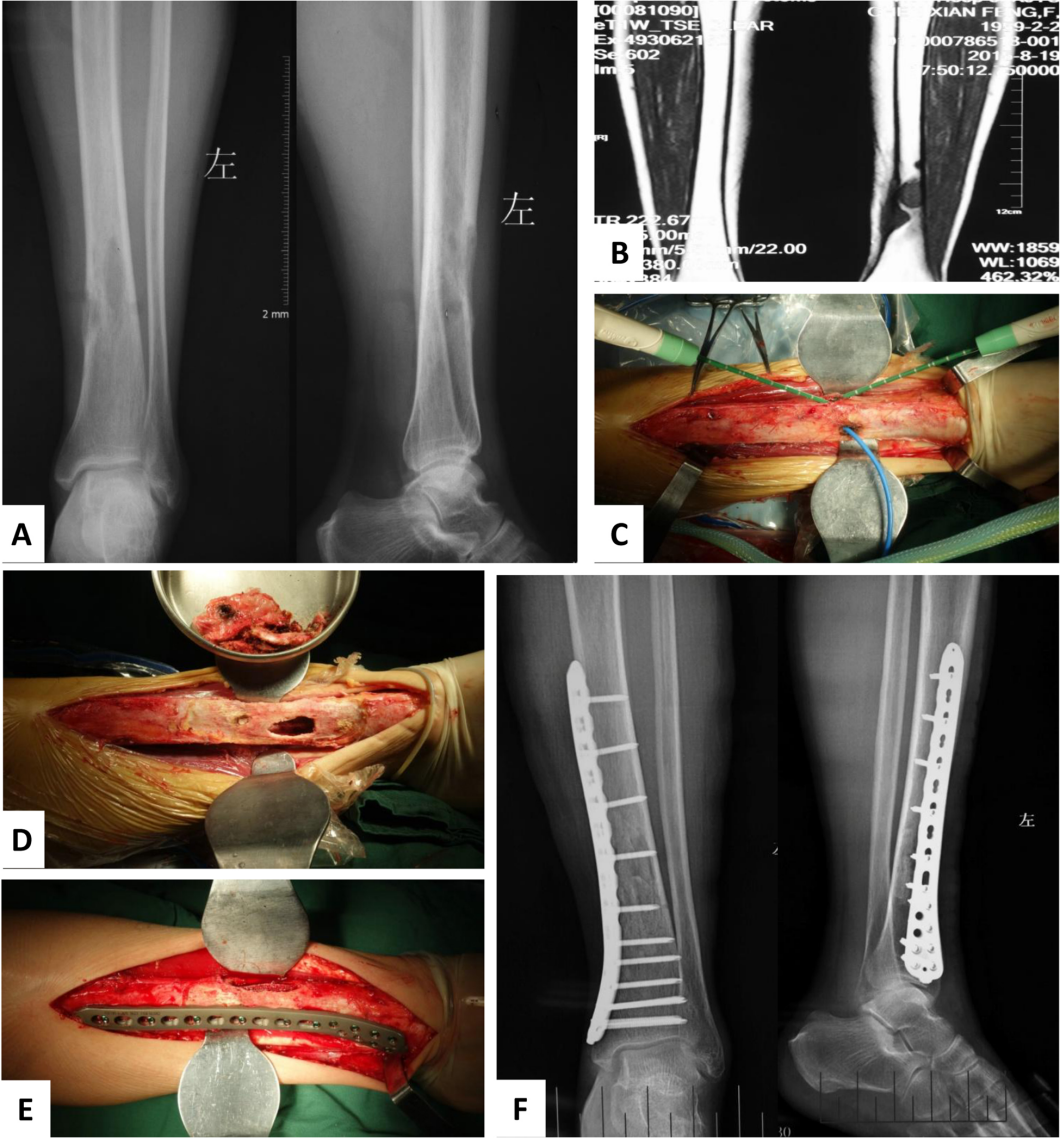
Typical procedure for malignant bone tumors at tibial diaphysis. **a** Image data of A-P and lateral X ray films showed a metastatic neoplasm from primary thyroid carcinoma to distal tibial diaphysis before surgery. The vague boundary of the bone defect demonstrated that the lesion might be malignant. **b** Preoperative MRI imaging film demonstrated the accurate location and involved range of the metastatic lesion. **c** The microwave generator and antenna were inserted into the tumor bulk and began to deliver electromagnetic energy into the tumor bone. **d** Tumor bone inside the tibial diaphysis was removed and bone graft cavity was prepared. **e** The allograft bone fragments were used to fill the bone defect and a prophylactic fixation with was conducted using molding steel plate. **f** Postoperative A-P X and lateral ray films demonstrated the tumor removal and tibia rebuilding

**Fig. 3:**
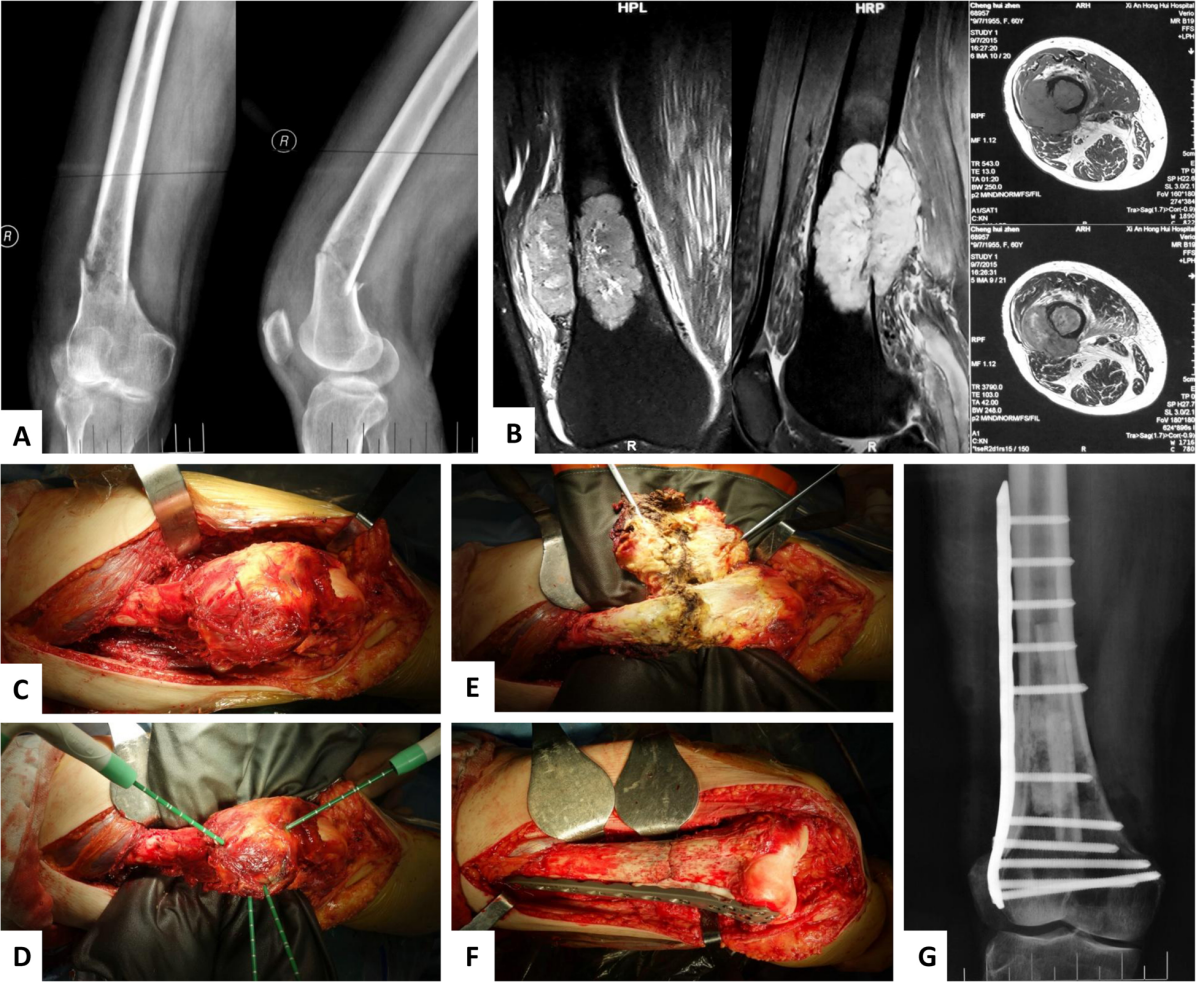
Typical procedure for pathological fracture from malignant bone tumors. **a** Image data of A-P and lateral X ray films showed a metastatic neoplasm from primary renal carcinoma to distal femoral diaphysis before surgery. There was visible bone destruction at the displaced fracture ends. **b** Preoperative MRI imaging film demonstrated the soft tumor mass and hematoma surrounding the metastatic bone lesion. **c** Dissect the tumor-bearing bone from surrounding normal tissues with safe margin without piercing the hematoma around the displaced fracture ends. To make sure the hematoma was not broken, the vastus intermedius was retained in situ and would experience microwave ablation process together with the lesion, soft tumor mass, and hematoma. **d** The microwave generator and antenna were inserted into the tumor bulk and began to deliver electromagnetic energy into the tumor bulk. **e** Soft tumor mass was removed and fracture ends were exposed. **f** The mixture materials of autologous fibular graft with bone cement were used for filling defect space and the fracture was reduced and fixed through rigid steel plate. **g** Postoperative A-P X ray film showed the perfect reduction and reliable fixation

With more than 20 years’ experience, we conducted typical MWA procedure and approaches for various parts of extremities and pelvis (Fig. 1). The incisions were required to be long enough, so that the adjacent vital neurovascular bundle could be carefully dissected and protected during ablation process. The surgeons must be familiar with the local anatomies because the osteotomy was seldom executed and the soft tissue surrounding the tumor mass was required to be accurately distinguished with reliable safe margins. The soft tissue coverage in the legs was relatively poor, and the local muscles and skin were supposed to be retained and protected in situ, besides neurovascular bundle (Fig. 2). In some cases, the incision safety could benefit from gastrocnemius muscle flap transfer. As some details for saving pathological fractures, the tumor-bearing bone should be dissected from surrounding normal tissues with safe margin without piercing the hematoma around the displaced fracture ends (Fig. 3). To make sure the hematoma was not broken, some adjacent muscles with secondary roles, i.e., the vastus intermedius in femoral diaphysis, would be retained in situ and experience microwave ablation process together with the lesion, soft tumor mass, and hematoma.

### Oncological results

**Fig. 4:**
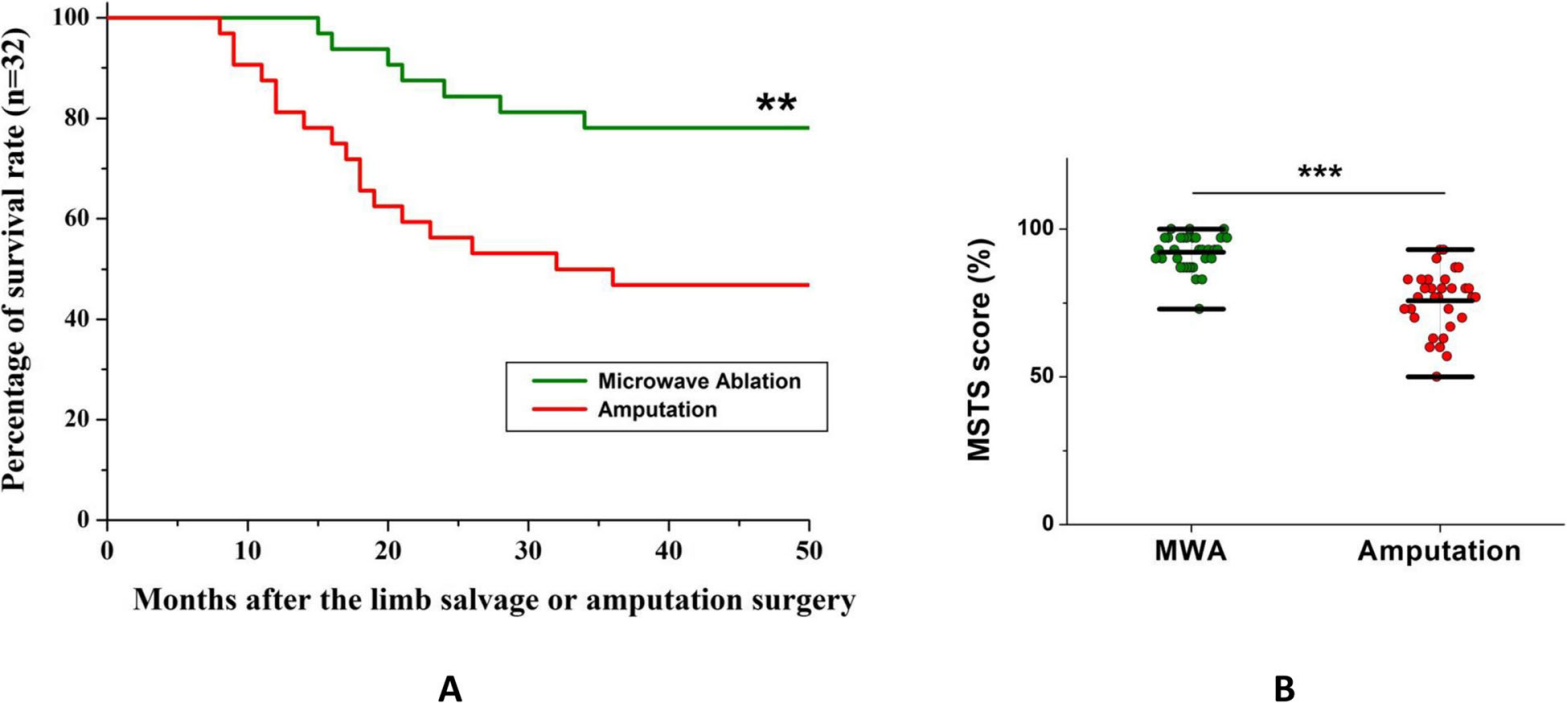
Comparison of clinical outcomes between the MWA group and amputation group. **a** The survival curve of diaphysis malignant tumors of femur/tibia treated with MWA verses amputation strategy. About 78% patients from the MWA population survived over 3 years after limb salvage surgery log-rank (Mantel-Cox) test compared to the amputation group (nearly 47%). Once 3 years follow-up achieved, no subsequent death occurred in both groups in this series study. **b** At the 6-month postoperative follow-up and the subsequent follow-up visits, patients were asked to attend an outpatient clinic where a survey of the MSTS was registered to assess patients’ function and satisfaction. The average postoperative MSTS rating score percentage was 92.13 ± 5.93%, which was significantly higher than the amputation group (75.81 ± 10.44%) in our consecutive inclusions. (*P < 0.05, **P < 0.01, ***P < 0.001)

Due to the good exposure and reliable protection of vital neurovascular bundle during the MVA procedure, 25 of 32 patients survived over 3 years and all of the patients alive have a satisfactory functional and cosmetic limb (Table 4). Six cases with high-grade malignancy died from cachexia, renal failure, or postoperative visceral metastasis, and one young patient with osteosarcoma died from chemotherapy reactions with the irreversible reduction of blood ternary systems (i.e., peripheral blood hemoglobin, white blood cell, and blood platelet count), during the third postoperative circle. The remaining 25 cases survived over 3 years with the longest follow-up period of 15 years. As a result of comparison, the postoperative survival rate of MWA group was significantly higher than the amputation group in consecutive inclusions (*P* = 0.0059) (Fig. 4a).

**Table 4:** Outcomes of patients of diaphysis malignant tumors of femur/tibia treated with microwave ablation

Types	No. of patients (percentage)
Alive or free of disease (≥ 3 years)	25 (78.125%)
Death	7 (21.875%)
Postoperative visceral metastases	3 (9.375%)
Cachexia	2 (6.25%)
Renal failure	1 (3.125%)
Reactions of chemotherapy	1 (3.125%)

### Functional results

All the patients alive had a functional and cosmetic limb. Most patients’ function was restored to sport level. At the final follow-up visit, no patients showed adjacent joint instability. The average postoperative MSTS rating score percentage was 92.13 ± 5.93%, which was significantly higher than the amputation group (75.81 ± 10.44%) in our consecutive inclusions (*P* = 0.00014) (Fig. 4b). We also reviewed some recent reports using different techniques to treat primary malignant musculoskeletal tumor, and the MWA therapeutic alternative has more advantages such as ablation in situ, reliable devitalization, less implants requirement, and less osteotomy requirement. The postoperative functional evaluation of MWA showed more superior outcomes than other therapeutic strategies, such as devitalizing autologous tumor-bearing bone in 99% alcohol for 30 min in vitro [10], in 20% sterile saline at 65 °C for 30 min [11], extracorporeal irradiation with X-rays [12], alcohol devitalization combined with prosthesis [13], prosthesis replacements [14], or other biological reconstruction after wide surgical resection [15].

### Complication

According to the MWA therapeutic principle, osteotomy was seldom required and nearly no prosthesis insertion was needed, therefore, the postoperative complication rate could be reduced to some level correspondingly (Table 5). Only one Ewing’s sarcoma patient was infected with gram-positive bacteria because of the poor soft tissue cover of proximal tibia, who got local control after debridement and benefit from gastrocnemius muscle flap transfer. Another vascular compromise was detected within 24 h postoperatively, and the patient with prostate metastatic neoplasm to distal femur was saved after the emergency vascular transplantation surgery. Three cases had recurrence, one was wrongly treated in other hospital by curettage so that the soft tissue contamination was too severe to get safe margin; the patient got local control after revision surgery. The other two cases suffered huge extraosseous tumor bulk, and amputation was needed after recurrence. Four cases with osteosarcoma suffered lung metastases and died within postoperative 2 years. Two postoperative fractures occurred within 2 years in consideration of the excessive ablation of the normal bone surrounding the lesion. Two cases respectively with very huge chondrosarcoma and osteosarcoma, osteotomy was needed during surgery to facilitate dissection, then delayed union occurred, one needs revision surgery.

**Table 5:** Complications of diaphysis malignant tumors of femur/tibia treated with microwave ablation

Types (of complication)	No. of patients (percentage)
Early complications	2 (6.25%)
Infection	1 (3.125%)
Vascular compromise	1 (3.125%)
Late complications	11 (34.375%)
Postoperative fracture	2 (6.25%)
Delayed union	2 (6.25%)
Local recurrence	3 (9.375%)
Remote metastases	4 (12.5%)

## Discussion

The use of high temperatures as treatment for multiple tumors, known as hyperthermic ablation, has existed for years [16]. Evidence regarding the therapeutically use of hyperthermic ablation for cancers of the liver, breast, and lung has been reported accumulatively [17, 18]. Microwave energy, typically at 2.45 GHz or 915 MHz, radiated by the antenna causes rapid agitation of water and other polar molecules in tissues, a process known as dielectric hysteresis. Therefore, microwaves enable faster heating of larger targets. During the procedure of involving thermal ablation, a set of thin ablation applicators were guided into the target tumor under image guidance. Electromagnetic energy was then applied into the tissue until temperatures rise to a cytotoxic level (50–60 °C), at which temperature, tumor cells around the applicators necrosed while the normal tissues outside of tumor sites preserved by cooling protection systems.

The main purpose of limb salvage surgery for malignant bone tumors is to save the diseased limbas far as possible through nomutilating surgery and without jeopardizing the prospects of survival; it has been performed for more than three decades. This type of surgical procedure mainly consists of two steps: en bloc resection of the tumor-bearing bone and reconstruction for the remained defect. Various techniques have been used for reconstruction, among which, metal prosthesis replacement has been widely used, and proved to be an effective practice [19]. Custom-made intercalary prosthesis has the advantage of allowing early weight-bearing training with adjuvant equipment and return to normal daily life. However, loosening, wearing, and breakage of prosthesis had been reported and the long-term survival rate was still of great concern, thereby limiting the use for young patients [20]. Compared with the patients with limited life expectancy, such as patients with metastatic lesion, whose early functional recovery is of greater concern than durability, but for children and adolescent patients, intercalary prosthesis is a less preferred option [21]. While MWA technique could be used to provide a relatively enough initial length of remained bones for further reconstruction and adequate mechanical strength for early weight-bearing training even only a little juxta articular bone is tumor free. The summarized pros-and-cons comparison of amputation, endoprosthesis, and MVA is available in Table 6.

**Table 6:** A summary of the pros and cons of surgical techniques for diaphysis malignant tumor of long bones

Surgical techniques	Pros	Cons
Amputation	Simple and time-saving technique. The cost is lower.	Compromised functional outcome and life quality.
Endoprosthetic reconstruction	Early functional recovery. Better cosmetic and psychological benefits. Life quality improved to some extent.	The cost is more expensive. The surgical challenge largely depends on bone defects. Prosthesis-related complications (infection, aseptic loosening, wearing peri-prosthetic fracture). High possibility of requiring revision surgery. Long-term functional outcome is controversial.
MVA	Long-term functional outcome and life quality largely improved. The cost is lower.	Need to be performed by well-trained surgical specialists.

If the traditionally defined wide margin resection was identified with intercalary resection technique, MWA technique could achieve a similar goal while retaining the curetted cortical bone intact (scaffold), thus making it easier and more durable for the following reconstruction of limb. If the devitalized bone scaffold together with fibular autograft and metal-ware implants, could endure in the first 1 or 2 years, it would thereafter achieve functional outcome for the duration of patient’s life because according to our experience, the devitalized bone can be gradually revitalized to living bone [22]. Once healing occurred, it is durable, so there is no worry about wear and tear as to the prosthesis.

In our previous study, it has been verified that MWA-processed tumor cells could be applied to induce specific antitumor effects [23]. In that paper, we mainly focused on the residual tumor cells from the “gray zone” of ablation, where the tissues and cells were likely to be incompletely ablated. Then we established the in situ ablation model, which is an ideal model for mimicking the MWA therapeutic process. After in situ ablation, the expression of Calreticulin (CRT), which is expressed on tumor cell surface and recognized as a critical role in antigen presenting process was significantly upregulated according to western blot and immunofluorescence assay. CRT was also one of the important signal molecules of immunogenic cell death [24]. The expression of CRT on the tumor cell surface might contribute to the tumor cell adhesion by dendritic cells and thus might elicit tumor-specific CD8^+^ T cell-mediated immune responses. These T cells might contribute to the elimination of survived tumor cells that have not been ablated directly by hyperthermia, leading to protect the patients from local recurrence or new metastasis. It was worth mentioning that the cell debris and degenerated organelles from whole ablated tumor cells might serve as general cancer antigens because the hyperthermia-induced organelle exposure of whole tumor cell compositions might be a superior alternative to other well-known antigen loading vaccines. In addition, these findings offer a feasible option for the use of MWA in combination with immunotherapy, especially for patients who have failed with chemotherapy or have limited options.

In summary, the present study provided valid evidence that MWA-based salvage limb surgery could be applied to provide a novel idea for malignant bone tumor treatment. Although MWA technique for treating malignant bone tumor is challenging for junior orthopedic surgeons, this surgery procedure could be mastered through persistent practice for years. According to our advice, it is worthy of such greatly persistent efforts to promote and popularize the MVA technique in limb salvage strategies for malignant bone tumor treatment. The early functional outcome is expected to be superior after MWA since neither prosthesis nor allograft could achieve better biocompatibility and biomechanical feature than an intact native material. Furthermore, the patients after MWA procedure could carry out earlier weight-bearing training and the long-term physical functions of reconstruction effected limbs might be more durable. Although this is a small size of case studies with relatively shorter follow-up time, our experience suggests that MWA might offer an innovative and distinctive therapeutic alternative for diaphysis malignant bone tumors.

## Data Availability

The data and materials are available from the department of orthopedic surgery, orthopedics oncology institute of Chinese PLA, Tangdu Hospital, Air Force Military Medical University. The datasets used and analyzed during the current study are available from the corresponding author on reasonable request.

## Abbreviations

MWA: Microwave ablation
MSTS: Musculoskeletal tumor society
CT: Computed tomography
